# Characterization of a 0.35T MR system for phantom image quality stability and *in vivo* assessment of motion quantification

**DOI:** 10.1120/jacmp.v16i6.5353

**Published:** 2015-11-08

**Authors:** Daniel L. Saenz, Yue Yan, Neil Christensen, Margaret A. Henzler, Lisa J. Forrest, John E. Bayouth, Bhudatt R. Paliwal

**Affiliations:** ^1^ Department of Medical Physics University of Wisconsin‐Madison Madison; ^2^ Department of Human Oncology University of Wisconsin‐Madison Madison; ^3^ Department of Surgical Sciences University of Wisconsin‐Madison Madison WI USA

**Keywords:** magnetic resonance imaging, image guidance, automatic contouring, motion tracking, adaptive radiotherapy

## Abstract

ViewRay is a novel MR‐guided radiotherapy system capable of imaging in near real‐time at four frames per second during treatment using 0.35T field strength. It allows for improved gating techniques and adaptive radiotherapy. Three cobalt‐60 sources (∼15,000 Curies) permit multiple‐beam, intensity‐modulated radiation therapy. The primary aim of this study is to assess the imaging stability, accuracy, and automatic segmentation algorithm capability to track motion in simulated and *in vivo* targets. Magnetic resonance imaging (MRI) characteristics of the system were assessed using the American College of Radiology (ACR)‐recommended phantom and accreditation protocol. Images of the ACR phantom were acquired using a head coil following the ACR scanning instructions. ACR recommended T1‐ and T2‐weighted sequences were evaluated. Nine measurements were performed over a period of seven months, on just over a monthly basis, to establish consistency. A silicon dielectric gel target was attached to the motor via a rod. 40 mm total amplitude was used with cycles of 3 to 9 s in length in a sinusoidal trajectory. Trajectories of six moving clinical targets in four canine patients were quantified and tracked. ACR phantom images were analyzed, and the results were compared with the ACR acceptance levels. Measured slice thickness accuracies were within the acceptance limits. In the 0.35 T system, the image intensity uniformity was also within the ACR acceptance limit. Over the range of cycle lengths, representing a wide range of breathing rates in patients imaged at four frames/s, excellent agreement was observed between the expected and measured target trajectories. *In vivo* canine targets, including the gross target volume (GTV), as well as other abdominal soft tissue structures, were visualized with inherent MR contrast, allowing for preliminary results of target tracking.

PACS number: 87.61.Tg

## I. INTRODUCTION

Modern radiotherapy delivers conformal dose distributions, but is limited by margins due to setup uncertainties and patient motion, both interfractional and intrafractional, leading to reduced tumor control probability (TCP) or the necessity of a larger CTV to PTV margin.^1^, ^2^, ^3^ Recent advances in image‐guided radiotherapy (IGRT), such as cone‐beam CT (CBCT) and implanted fiducials, are imaging modalities which alert clinicians of such motion before or during treatment. Another modality is magnetic resonance imaging (MRI), which has expanded IGRT recently with ViewRay (ViewRay Inc., Oakwood, OH), a commercially available radiotherapy treatment modality providing MR imaging in user‐defined planes during irradiation. Other MR‐guided radiotherapy projects are also under investigation, such as the MR‐linac in Utrecht integrating a 6 MV linear accelerator with a 1.5 T MR scanner.^4^, ^5^, ^6^ Progress in MR‐delineated autocontouring for lung tumors has also been made with a simulation of MR images at 0.2–0.5T, suggesting the ability to track lung tumors in low‐field MR linacs.^(7)^ With ViewRay, however, patients are already under treatment at a handful of institutions, and live motion previously unrecorded is being revealed.^8^, ^9^, ^10^, ^11^

ViewRay consists of three Co‐60 sources (∼15,000 Ci) in heads separated by 120° with concurrent MR imaging by a 0.35 T magnetic field. The magnet is maintained by a split magnetic bore system, while the treatment gantry rotates between the magnetic bores. Step‐and‐shoot IMRT delivery is made possible by the multileaf collimators (MLCs) on each head, each with two banks of 30 leaves. The imaging made possible by ViewRay is used in an online beam control approach which tracks targets and gates the radiation beam on or off, depending on the target position relative to a predefined allowable range of target positions. A target is specified and its contour generated during the daily three‐dimensional setup MR used for positioning and dose prediction. Next, the user selects or defines a region‐of‐interest surrounding the target in which the target is allowed during treatment. Once the beam is initiated, the target is automatically contoured every quarter of a second or half a second (for one or three slices, respectively). If this contour encroaches upon the boundary of the allowed region, the radiation beam will be turned off. Furthermore, a certain percentage of the target can be allowed to exit the allowed region before beam interruption, if desired. Alternatively, a time interval can be specified for a return to the treatable region before beam shut‐off.

The primary objectives of this study were to characterize the MR imaging parameters and their stability, and to validate the image capabilities for tracking. MR imaging parameters include signal‐to‐noise ratio (SNR), contrast, and spatial resolution. The physical accuracy of the imaging capabilities were also tested in a motion phantom. These tests were intended to investigate spatial integrity, as distortion, for example, is a formidable issue in radiation therapy due to the possible incorrect mapping of objects in the reconstructed image reducing the accuracy of dose calculation.^12^, ^13^ ViewRay corrects for image distortion across the field of view, and the accuracy was evaluated here through the motion phantom study. Finally, *in vivo* organ motion assessment in canines has been performed as a validation of the imaging workflow and a presentation of *in vivo* motion quantification. The built in autocontouring and beam gating is left to future studies, as we focus on the imaging alone by testing image stability and automatic tracking with an independent tool.

## II. MATERIALS AND METHODS

### A. Phantom studies

MR imaging characteristics of the ViewRay system were assessed using the American College of Radiology (ACR) phantom and its protocol (http://www.acr.org) which includes recommended acceptance criteria for clinical sequences (Table 1).^14^, ^15^ Images of the ACR phantom were acquired using a head coil (which conforms best to the phantom for reproducibility), following the ACR scanning instructions. Both ACR T1 and T2‐weighted sequences made available in the MR‐only mode were acquired. The scan time for the T1 and T2‐weighted sequences were 53 min and 76 min, respectively. These scans included 25 and 9 averages, respectively, to account for the relatively low signal in a 0.35 T magnet. The high number of averages (resulting in long scan times) has been established for ACR phantom testing with ViewRay in a previous study.^(16)^ While these sequences are available to the user in MR‐only mode for functional testing of the machine, the clinically available sequences, in fact, have a combination of T1 and T2 weightings. Measurements were performed monthly over a period of seven months (with an additional two instances in the first month). In all, nine ACR measurements are performed. The images were analyzed and the results were compared with the ACR acceptance levels.

### B. Motion phantom studies

The first study of our ability to image and measure motion was performed in a phantom setting. A cylindrical MR‐compatible phantom with an open end was designed. The target subject to motion was a silicon di‐electric gel poured into a beaker. Motion was provided by the motor from the QUASAR Respiratory Motion Phantom (Modus Medical Devices Inc., London, Canada), a phantom designed for quality assurance involving moving targets (e.g., 4D CT, respiratory gating). A plastic rod connected the target to the drive assembly. The motor was situated in the vault with the magnetic field present at all times. As the motor should not interfere with MR imaging nor should the magnetic field complicate the motor's operation, the motor was placed at the end of the treatment couch where the magnetic field is minimized. To mitigate RF noise from the motor, it was securely fastened in a wooden box, wrapped on the inside and outside with copper foil to create a Faraday cage. A wire was connected to ground electric currents. The target was driven in a sinusoidal motion pattern in a single dimension with total peak to trough displacement of 40 mm. The duration of each cycle (cycle length) was varied from 3 to 9 s per cycle (in 1 s increments).

Images were acquired in the sagittal orientation in one plane along the axis of the cylindrical target with known area of 3.9 cm^2^. The imaging sequence tested here is the TrueFISP pulse sequence, capable in general of rapid imaging of six frames per second.^(17)^ In the ViewRay iteration, four frames per second are possible. TrueFISP employs a balanced gradient waveform which returns signal to the same phase it had before starting a gradient sequence. A 27cm×27cm field‐of‐view (FOV) was utilized with a 1491 Hz/pixel bandwidth and 3.5×3.5×10.0 mm pixel size. The TR was 2 ms with a TE of 0.86 ms. Two averages were used to form each frame. In the current treatment planning and delivery software, cine MR imaging (rapid frame rate planar imaging) was only possible in the treatment mode. Therefore, a mock treatment plan was created for the purpose of enabling treatment delivery to allow for imaging.

An independent morphological thresholding‐based automatic segmentation software developed at our institution processed the images to locate the target.^(18)^ A binary image was created with empirically determined thresholds. Morphological processing via erosion and dilation isolated the target from random noise in the image and restored the object to its original size. The centroid position of each contour was used as an estimate of target position in each frame of the MR images. The centroid positions along the direction of motion were fitted to a sinusoidal shape using the MATLAB curve fit tool (MathWorks, Natick, MA) to test the ability of the imaging system to accurately portray target motion. In the equation of fit, an amplitude (A), angular frequency (B), phase (C), and background term (D) were allowed (y=A sin(Bx+C)+D). The curves were assessed for goodness of fit by the sum of squared residuals, R‐square, adjusted R‐square, and root‐mean‐square error.

### C. Canine patients

Phantom studies were followed up with *in vivo* imaging in canine subjects as part of a study to characterize the extent of motion visible in CT and MR. Four dogs were subject to pre‐ and postcontrast CT, 4D CT, and ViewRay 3D/cine imaging. Subjects were anesthetized and exhibited free breathing. They were localized in Vac‐Lock bags (Elekta Medical Intelligence, Atlanta, GA) which conform to the canine patient to aid with patient setup. All imaging was performed in the same evening. Six sites (two sites were examined in two of the animals) were indicated by the veterinary resident for study based on his priorities for the animal's care and interest in local motion. Though two animals contributed multiple sites to the study, the distinct sites represented regions of varying MR contrast, enabling a wider range of targets for study at the cost of independence between the six sites. After completion of the 4D CT, the subjects were transported to ViewRay while a treatment plan for the purposes of imaging only was devised. The canines received no radiation dose as the unit had not yet received the Co‐60 sources at the time of the study. A 3D pilot volume, high resolution volume, and cine based on the same TrueFISP sequence described above were performed. The impact of the residual nonparamagnetic CT contrast was not considered since contrast was already diminished at the time of the 4D CT (a few minutes postinjection) compared to the MR imaging (an hour and a half postinjection). Furthermore, contrast would be concentrated in the urinary tract at the time of ViewRay scanning. The acquired sagittal slices were chosen by the veterinary resident based on maximizing the inclusion of organs subject to motion. The sites chosen for contouring (liver, kidney, gross target volume) were those of clinical interest to the veterinarians and those sufficiently characterized for the automatic morphological processing contouring algorithm to process. Resultant cine images were exported into video which was loaded into MATLAB for processing.^(19)^ Automatic contours were then obtained from the morphological processing based tool described above.

To supplement the organ motion assessment found by the automatic approach, manual contouring was also performed. The manual contours were generated by hand under the direction of the veterinary resident. Once obtained, the manual and automatic trajectories were acquired equivalently, by using the contour centroid as a marker of position.

Agreement between the two techniques would lend credibility to the results as they would be obtained in two different ways. Therefore, the two sets of contours (manual and automatic) were then compared via the Dice similarity coefficient (a measure of contour similarity based on overlap). The correlation between contour centroid positions via the two methods was also calculated, and a two‐sided Student's paired *t*‐test testing the null hypothesis of no correlation was performed. Other criteria included the sensitivity (the fraction the manual contour in the automatic contour) and the positive predictive value (the fraction of the automatic contour in the manual contour). Finally, the modified Hausdorff distance, an average distance between points along a contour, was calculated.^(20)^ Mean and maximum deviations in target position were analyzed as well, as has been examined previously in the literature.^(21)^ Finally, the correlation coefficient between the two methods was examined.

## III. RESULTS

### A. Phantom studies

A summary of the ACR test results is given in Table 1. Mean values for each ACR MRI phantom measurement was within the acceptable limits for the T1 and T2 ACR scan protocols available in the MR‐only mode. Minimum and maximum results for each test are also presented in Table 1, where it is shown that, for two of the T1 distance measurements and two slice position accuracy tests, a maximum value was outside of tolerance, but average results were well within the limits.

**Table 1:** Results and statistics of the ACR phantom analysis results over nine time points using the head coil. Results are shown for the T1‐ and T2‐weighted ACR scanning protocols provided by ViewRay in the MR‐only mode

*Test*	*Tolerance*	*Average Value*	*SD*	*Min. Value*	*Max. Value*
Localizer Geometry Accuracy (mm)	148±2	147.5	0.9	146.5	148.9
*T2 Image Test*					
High Contrast Spatial Resolution (mm)					
Upper Left	≤1.0	0.9	0.04	0.9	1.0
Lower Right	≤1.0	0.9	0.04	0.9	1.0
Slice Thickness Accuracy	5.0±0.7	5.0	0.3	4.6	5.5
Slice Position Accuracy (mm)					
Slice 1	≤5	0.0	1.4	−2.8	2.1
Slice 11	≤5	−1.8	0.9	−3.9	−0.7
Percent Signal Ghosting	<0.025	0.0004	0.0005	0.0	0.001
Low Contrast Detectability	≥9	22.3	3.3	17	25
*T1 Image Test*					
Geometry Accuracy (mm)					
Slice 1					
Top/Bottom Distance	190±2	190.0	0.7	189.0	190.9
Left/Right Distance	190±2	190.6	0.5	190.1	191.4
Slice 5					
Top/Bottom Distance	190±2	190.7	0.6	189.8	192.0
Left/Right Distance	190±2	190.4	0.5	189.6	191.3
Top Left/Bottom Right Distance	190±2	190.5	1.7	189.6	195.2
Top Right/Bottom Left Distance	190±2	191.4	1.9	189.2	196.6
High Contrast Spatial Resolution					
Upper Left	≤1.0	0.9	0.04	0.9	1.0
Lower Right	≤1.0	0.9	0.04	0.9	1.0
Slice Thickness Accuracy (mm)	5.0±0.7	5.0	0.3	4.5	5.5
Slice Position Accuracy (mm)					
Slice 1	≤5	0.5	2.1	−2.5	5.2
Slice 11	≤5	−0.8	2.7	−3.7	6.4
Image Intensity Uniformity	>87.5%	91.8%	2.5%	87.6%	94.4%
Percent Signal Ghosting	<0.025	0.015	0.007	0.001	0.022
Low Contrast Detectability	≥9	28.6	3.5	23	33

### B. Motion phantom studies

**Figure 1:**
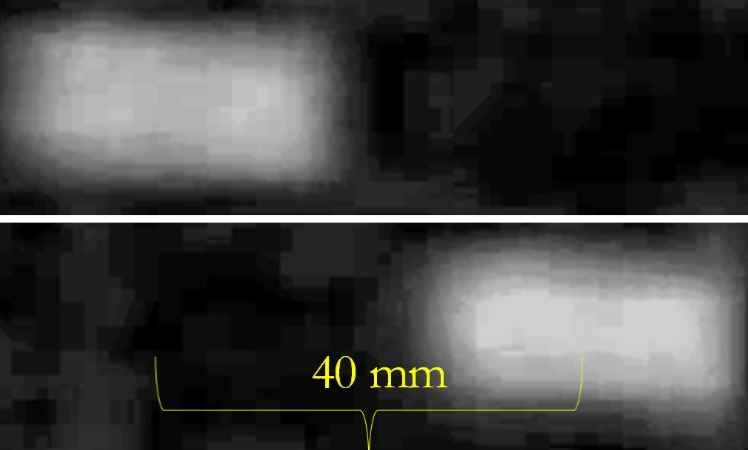
Visualization of a cylindrical target along its long axis at the thickest region during target motion imaged with 0.25 s temporal resolution. The two images show both ends of the sinusoidal trajectory.

**Figure 2:**
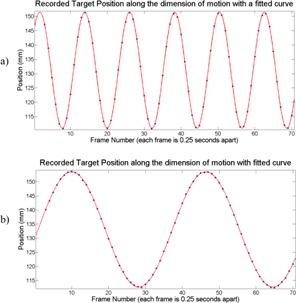
The measured target position along the dimension of motion in each frame. The automatic contour's centroid was used as a mark of position in each frame. The solid curve represents the sinusoidal curve fit generated with MATLAB. Figure 2(a) shows the motion quantification for the most rapid trajectory (3 s per cycle), while Fig. 2(b) represents the slowest trajectory (9 s per cycle). The target was driven at an amplitude of 20 mm.

**Figure 3:**
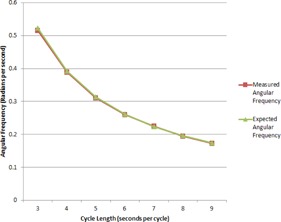
The expected angular frequency of simulated target sinusoidal motion is plotted, along with the measured based on segmentation of the images. The expected angular frequency is calculated as 2π/T, where T is the known period of motion. The measured result comes from the curve fit.

Target motion was clearly visualized in the resulting images. Over the range of 3 to 9 s per cycle, the frame rate provided by ViewRay was sufficient to qualitatively visualize target motion. Figure 1 indicates example images of the target at both ends of its motion. Figure 2 plots the trajectory of the target along the dimension of motion with a fit for the most rapid three seconds per cycle motion (Fig. 2(a)) and the slowest 9 s per cycle motion (Fig. 2(b)). Each data point represents the component along the direction of motion of the automatic contour centroid coordinate. Across the range of frequencies, excellent agreement is observed. Table 2 demonstrates the sinusoidal fitting parameters for all target rates. For the 7 s per cycle curve fit, a data point seemed to be skipped in the motion quantification, possibly due to rounding in time if the MR frames are not precisely 0.25 s apart. This led to the slightly larger sum of squared residuals. The R‐square value suggests excellent agreement nevertheless. Figure 3 plots the measured angular frequency from the curve fit along with the theoretical angular frequency calculated from 2π/T, where T is the period of motion (in units of frame numbers).

**Table 2:** For each of the cycle lengths at which the simulated cylindrical target was driven, the resulting images were segmented and the centroid positions plotted against frame number were fitted to a sinusoidal curve. The fitting parameters from the MATLAB sinusoidal curve are presented here. The target was driven with a 20 mm amplitude at 3–9 s per breath

*Cycle Length (s per breath)*	*Amplitude (mm)*	*Measured Angular Frequency (radians/s)*	*Sum of Squared Residuals*	*R‐square*	*Adjusted R‐square*	*Root‐mean‐square Error*
3	20.26	0.516	1.99	0.9999	0.9999	0.176
4	20.36	0.389	1.21	0.9999	0.9999	0.131
5	20.37	0.311	1.22	0.9999	0.9999	0.137
6	20.42	0.259	1.85	0.9999	0.9999	0.169
7	20.17	0.225	46.1	0.9966	0.9964	0.848
8	20.35	0.195	1.70	0.9999	0.9999	0.163
9	20.45	0.173	1.37	0.9999	0.9999	0.147
Average	20.34					
SD	0.096					

### C. Canine patients

**Figure 4:**
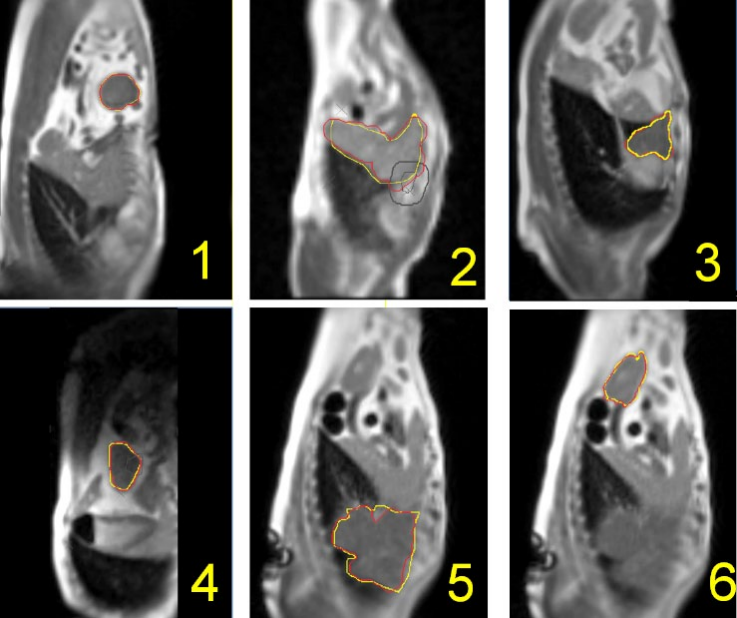
Example cine images (acquired with temporal resolution of 0.25 s) for the six sites studied. Morphological‐based automatic contours are shown in red, while manual contouring is demonstrated in yellow.

**Figure 5:**
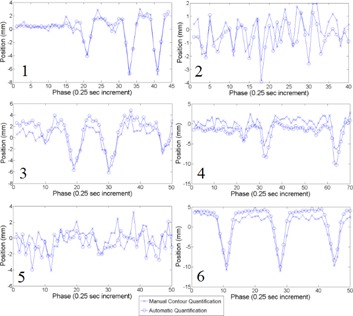
Trajectories of the same structures as for Fig. 4 in the craniocaudal direction. Data points are the centroid of the automatic contour (the craniocaudal coordinate). Results are shown for manual and automatic contours.

Figure 4 demonstrates the contouring (both automatic and manual) on sample images for the canines. The liver, kidneys, stomach, and bowel among other abdominal organs were clearly visible in the cine imaging, suggesting the ability to contour partially based on image contrast. Temporal resolution in the images was sufficient to visualize the beating heart and to observe cardiovascular pulsatility in larger vessels, as well. Figure 5 demonstrates the trajectories the organs were subject to, determined manually and via the independent automatic contouring approach.

Table 3 shows the mean and maximum deviation between the automatic and manual motion quantification methods across the frames used. The mean difference in all cases was under 2 mm. Furthermore, similarity metrics including sensitivity was high at 0.88 (Table 4). The Dice similarity coefficient averaged to >0.9, showing a high amount of overlap. The PPV was 0.95. The modified Hausdorff distance mean value of 2.5 mm indicates that degree of proximity between contour boundaries. Table 5 demonstrates correlation coefficients (twelve total for the two dimensions in the six sites) and the p‐value from a two‐sided paired Student's *t*‐test tested against the null hypothesis of no correlation. Of the 12 correlation coefficients presented in Table 5, seven measurements are deemed high correlation coefficients (defined as greater than 0.6). Three measurements exhibited more moderate correlation (0.3 to 0.6), and only two exhibited a weak correlation coefficient of less than 0.3. Moreover, 10 of them are statistically significant with a p‐value <0.05. In the two cases in which the null hypothesis failed to be rejected, little motion existed (anterior–posterior dimension of motion).

**Table 3:** The mean and maximum deviation across all frames between two motion quantification methods (manual/automatic) for the six canine imaging sites. Position in both dimensions is determined by the centroid position of the contours

		*Craniocaudal Difference (mm)*		*Anterior–Posterior Difference (mm)*	
*Site Number*	*Site Type*	*Mean*	*Maximum*	*Mean*	*Maximum*
Site 1	GTV	0.4	1.3	0.5	1.5
Site 2	Liver	0.9	2.6	1.5	3.5
Site 3	GTV	1.3	2.5	1.0	3.6
Site 4	Kidney	1.9	4.0	1.1	2.1
Site 5	GTV	1.2	3.9	1.4	3.6
Site 6	Kidney	1.6	3.7	1.4	2.2
Average		1.2	3.0	1.2	2.8

**Table 4:** Similarity metrics averaged across imaging frames between the manual and automatic contour generation approaches. The ± in the final row indicates the mean ± standard deviation (SD) across the sites

		*Dice Similarity Coefficient*		*Sensitivity*		*Positive Predictive Value*		*Modified Hausdorff Distance (cm)*	
*Site Number*	*Site Type*	*Mean*	*SD*	*Mean*	*SD*	*Mean*	*SD*	*Mean*	*SD*
Site 1	GTV	0.94	0.01	0.89	0.03	0.99	0.01	0.17	0.04
Site 2	Liver	0.89	0.01	0.90	0.02	0.88	0.02	0.27	0.03
Site 3	GTV	0.91	0.02	0.90	0.02	0.93	0.03	0.22	0.04
Site 4	Kidney	0.91	0.02	0.85	0.03	0.99	0.01	0.23	0.04
Site 5	GTV	0.92	0.01	0.88	0.02	0.96	0.02	0.41	0.05
Site 6	Kidney	0.92	0.01	0.87	0.02	0.98	0.01	0.21	0.03
Average		0.91±0.01		0.88±0.02		0.95±0.04		0.25±0.08	

**Table 5:** The correlation between the position estimates of the two contouring methods in the two dimensions of motion was investigated, and the correlation coefficients are presented for each of the six sites

		*Correlation Coefficient*		*p‐value Testing Null Hypothesis of no Correlation*	
*Site Number*	*Site Type*	*Craniocaudal*	*Anterior–Posterior*	*Craniocaudal*	*Anterior–Posterior*
Site 1	GTV	0.97	0.39	$<1\times 10^{-10}$	$9.5\times 10^{-3}$
Site 2	Liver	0.63	0.22	$1.2\times 10^{-5}$	0.17
Site 3	GTV	0.96	0.50	$<1\times 10^{-10}$	$<1\times 10^{-10}$
Site 4	Kidney	0.92	0.90	$<1\times 10^{-10}$	$<1\times 10^{-10}$
Site 5	GTV	0.37	0.04	0.01	0.8
Site 6	Kidney	0.98	0.62	$<1\times 10^{-10}$	$1.3\times 10^{-6}$

## IV. DISCUSSION

Imaging characteristics, exemplified by the ACR phantom analysis procedure, were consistent, with repeatable passing results. Performance did not systematically vary across the time period investigated. This stability indicated reliable imaging ability of a quality sufficient for radiotherapy guidance upon which to build an image‐guided radiotherapy program. The numerical results presented in Table 1 will serve as important baselines in order to design monthly quality assurance procedures, including action levels and test frequencies. Other institutions interested in the imaging quality of ViewRay can now better understand the quantitative abilities via the industry‐wide ACR MRI phantom tests. The ACR scans are not directly applicable to the clinically available scans given their long scan times compared to the rapid imaging required for patient setup. Nevertheless, these tests help identify performance changes in system components, including the imaging coils or shim coils for the maintenance of magnetic field homogeneity. Drawbacks of the 0.35 T imager included minor breakdown in the steady‐state MR signal established with the TrueFISP sequence, which occasionally appeared as dark, curved bands across the image near the corners. TrueFISP being the only pulse sequence clinically available also limits the scope of the technology for the time being, but does meet the radiotherapy need for rapid visualization instead of high‐contrast or high‐resolution tumor imaging. Geometric accuracy, though difficult to ensure in MR, was confirmed within 1 mm over the central 20 cm diameter sphere and within 2 mm over the central 35 cm diameter sphere.

The phantom experiments demonstrated that the cine images were true to the real motion of the phantom over the periods of 3 to 9 s for motion amplitudes of 40 mm. Quantitatively, the trajectories fitted to sinusoidal motion with a background term agreed very well with the known motion parameters. Physical timing was preserved in the motion output with little temporal inaccuracy in the measured positions. Each parameter in the curve fits indicated good agreement, with the amplitude of motion measured accurately (to within 0.3 mm agreement on average with the known 20 mm amplitude, an accuracy of better than 2%). Measured and expected angular frequency versus target rate curves laid directly upon one another. In only one case (7 s per cycle) was the R‐square value anything under 0.9999. These results indicate a degree of confidence a user can have in the temporal and spatially accurate representations ViewRay exports in its images as they are applied to image‐guided radiotherapy.

*In vivo* images were examined for sufficient quality to automatically extract motion information. Doing so with an independent automatic contouring approach demonstrated the possibility of real‐time motion quantification. An agreement with manual contouring indicates that these rapidly obtained contours are similar to those that would be obtained manually. Therefore, we are more confident in the motion quantification, since it was measured independently via two methods. The results presented here are, nevertheless, preliminary with four canine subjects and represent a first iteration in the process of characterizing the tracking capabilities of the ViewRay MR‐imaging component. The reproducibility of the results requires further investigation, which will come with future animal imaging studies and human patients. Of course, the canine imaging here will not translate directly to human imaging. The larger size of a human patient can result in loss of signal far from the MR coils near the center of the body. Furthermore, imaging under anesthesia is not representative of human patients undergoing radiotherapy. Nevertheless, the initial images are promising for potential IGRT with human patients.

Initial experiences with the workflow (a major purpose of canine study) validate the integration of imaging and therapy provided by ViewRay. Future studies might present a similar study with human subjects, who will benefit from not only the increased experience with ViewRay workflow, but from the knowledge of which organs can be expected to be visualized.

## V. CONCLUSIONS

The images acquired with ViewRay demonstrated a consistent performance, as well as an accurate visualization of target motion, both in‐phantom and *in vivo.* Image performance did indeed pass the ACR criteria. This quantitative assessment of image quality translated to images representing the physical trajectories underwent by a test object under controlled conditions. The ViewRay system successfully captured motion in this case.

In the canine studies, targets including GTV, as well as other abdominal soft tissue structures, benefitted from MR contrast and were capable of clear segmentation. The ability alone to create automatic contours indicated a suitable degree of image contrast for target localization, a positive result for the underlying imaging as we move forward toward investigating the built‐in automatic contouring in ViewRay. Near real‐time imaging during treatment is of sufficient spatial accuracy and temporal resolution to track targets as they move, and suggests the ability for superior visualization in motion management.
